# Targeting Artificial Tumor Stromal Targets for Molecular Imaging of Tumor Vascular Hypoxia

**DOI:** 10.1371/journal.pone.0135607

**Published:** 2015-08-26

**Authors:** Nathan A. Koonce, Joseph Levy, Matthew E. Hardee, Azemat Jamshidi-Parsian, Kieng B. Vang, Sunil Sharma, James A. Raleigh, Ruud P. M. Dings, Robert J. Griffin

**Affiliations:** 1 Department of Radiation Oncology, Winthrop P. Rockefeller Cancer Institute, University of Arkansas for Medical Sciences, Little Rock, Arkansas, United States of America; 2 Center for Integrative Nanotechnology Sciences, University of Arkansas at Little Rock, Arkansas, United States of America; 3 Department of Radiation Oncology, University of North Carolina School of Medicine, Chapel Hill, North Carolina, United States of America; Center for Interdisciplinary Research in Biology (CIRB) is a novel Collège de France / CNRS / INSERM, FRANCE

## Abstract

Developed and tested for many years, a variety of tumor hypoxia detection methods have been inconsistent in their ability to predict treatment outcomes or monitor treatment efficacy, limiting their present prognostic capability. These variable results might stem from the fact that these approaches are based on inherently wide-ranging global tumor oxygenation levels based on uncertain influences of necrotic regions present in most solid tumors. Here, we have developed a novel non-invasive and specific method for tumor vessel hypoxia detection, as hypoxemia (vascular hypoxia) has been implicated as a key driver of malignant progression, therapy resistance and metastasis. This method is based on high-frequency ultrasound imaging of α-pimonidazole targeted-microbubbles to the exogenously administered hypoxia marker pimonidazole. The degree of tumor vessel hypoxia was assessed in three mouse models of mammary gland carcinoma (4T1, SCK and MMTV-Wnt-1) and amassed up to 20% of the tumor vasculature. In the 4T1 mammary gland carcinoma model, the signal strength of α-pimonidazole targeted-microbubbles was on average 8-fold fold higher in tumors of pimonidazole-injected mice than in non-pimonidazole injected tumor bearing mice or non-targeted microbubbles in pimonidazole-injected tumor bearing mice. Overall, this provides proof of principle for generating and targeting artificial antigens able to be ‘created’ on-demand under tumor specific microenvironmental conditions, providing translational diagnostic, therapeutic and treatment planning potential in cancer and other hypoxia-associated diseases or conditions.

## Introduction

Although initially thought to be homogeneous, early proof of heterogeneous physiology in tumor vessels were described with low oxygen tensions (<10 mmHg pO_2_) found in selected tumor blood vessels [1–5]. This low oxygen or hypoxic environment leads to stabilization of the transcriptional regulating protein hypoxia inducible factor (HIF-1α), meditating downstream signaling of pro-angiogenic proteins and small molecules which have been implicated in driving angiogenesis and malignant progression [6, 7]. In recent years, the hypoxic, perivascular niche has been extensively studied and demonstrated to harbor cancer stem cells, as well as generally promoting metastatic spread [8–11]. However, the majority of these investigations have been largely *in vitro* or focused on genetically manipulated HIF-1 protein knockout mouse models [6, 7] where the true details of hypoxia and cell type(s) involved are not well documented.

In addition to hypoxia-related tumor progression, hypoxia-induced protection against standard cytotoxic therapies leading to suboptimal response and eventual tumor recurrence has been well documented [12–14]. Mounting evidence suggests high-dose radiotherapy response in solid tumors is mediated by indirect death resulting from direct radiation-induced tumor endothelial cell death [15]. A low pO_2_ environment protects cells from radiation-induced cell death by reducing the oxygen fixation of DNA strand breaks. In the presence of O_2_, free radicals formed following ionizing radiation potentiate DNA damage resulting in cellular death while hypoxic environments attenuate this effect [16]. Applying hypoxic radioprotection dogma to the idea of vascular-damaging doses of radiation or other vascular-targeted therapies is a field in large part unstudied. Despite compelling evidence demonstrating the impact of the hypoxic tumor vessel niche on tumor progression, clinical techniques evaluating the oxygenation level in tumor vessels or tumor endothelial cells themselves remains elusive, limiting the ability to study the impact on therapeutic response.

The bioreductive compound pimonidazole is a substituted 2-nitroimidazole that is preferentially reduced in viable hypoxic cells forming an artificial tumor target that can be detected by flow cytometry and immunohistochemistry [17]. Irreversible reduction of pimonidazole allows cells which are intermittently hypoxic to also be detected by the aforementioned techniques, and thus reoxygenation is not a limiting factor. Unlike naturally occurring tumor-associated antigens [18], hypoxia marker antigens are not susceptible to the inherent heterogeneous tumor cell genetics or changes in protein expression and genetic drift during disease progression or therapeutic intervention. These aspects coupled with the observation of pimonidazole dependent complement cell lysis suggesting pimonidazole antigens exist on the cell membrane led to the current investigation [19]. Therefore, in the present study, we tested the utility of pimonidazole antigen-like targets formed in the luminal surface of tumor blood vessels as a target for intravenously-injected molecular contrast agents that may avoid many of the pitfalls that face traditional hypoxia imaging and quantification. We describe a novel hypoxemia (vascular hypoxia) detection method based on high-frequency ultrasound imaging of vascular restricted α-pimonidazole targeted-microbubbles, targeting the hypoxia marker pimonidazole in mouse mammary gland carcinoma models.

## Materials and Methods

### Cell lines

Murine endothelial cells (2H11) and murine breast carcinoma cells (4T1 & SCK) were cultured at 5% CO_2_ and 37°C. Cell lines were maintained in the following media conditions: 2H11—DMEM high glucose (4.5 g/L) + 10% FBS, 4T1—DMEM F-12 (Gibco) + 10% BCS, and SCK—RPMI1 1640 + 10% BCS. 2H11 cells were purchased from ATCC, 4T1 cells were obtained from Dr. Michael Borrelli (UAMS) and SCK cells were obtained from Dr. Chang Song (U of Minn.) [20, 21]. All cell lines were tested and negative for mycoplasma.

### Generating pimonidazole-targeting microbubbles

**Fig 1:**
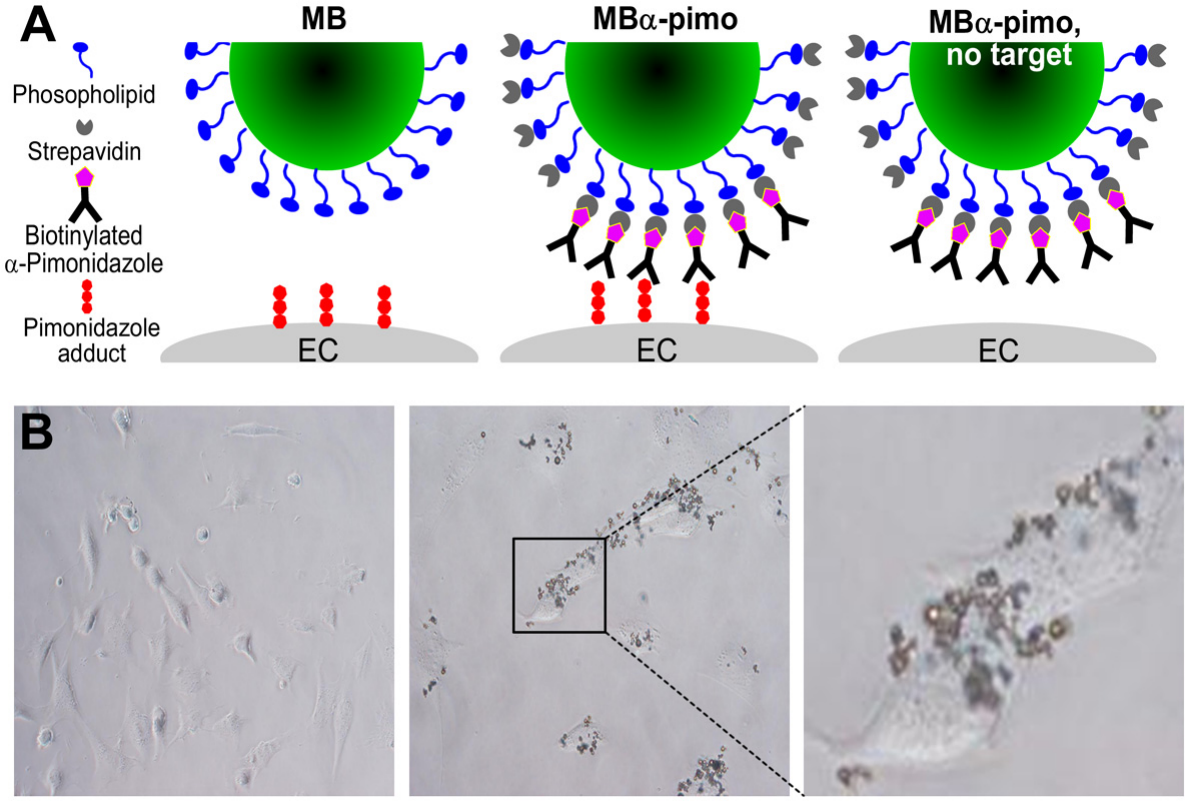
Pimonidazole targeting microbubbles. **(A)** A graphic representation of the microbubbles and conditions used. *Left*, unlabeled microbubbles (MB); middle, pimonidazole-targeting MB (MBα-pimo); and *right*, MBα-pimo without pimonidazole present in the circulation. **(B)** MBα-pimo binds hypoxic 2H11 endothelial cells only in the presence of pimonidazole. MBα-pimo does not bind endothelial cells (*left*), unless pimonidazole is added (*middle*). (*right*), MBα-pimo binding to the cell surface of hypoxic endothelial cells, magnification 40X.

Commercially available microbubbles, on average 2.5 μm in diameter with 1 x 10^5^ streptavidin biding sites, allow for molecular targeted imaging (FUJIFILM Visualsonics, Inc.) [1]. In brief, streptavidin molecules coat a lipid shell containing a gas filled hollow core of perfluorobutane/nitrogen (C_4_F_10_/N_2_). Here, 20 μg of biotinylated anti-pimonidazole antibody [22] was incubated for 15 minutes with streptavidin-coated microbubbles to create pimonidazole targeting microbubbles, MBα-pimo (stock concentration 2 x 10^9^/mL) (Fig 1A). Microbubbles are stable up to 3 hours after preparation and have a clearance rate of 10–20 minutes in mice.

### *In vitro* microbubble binding experiments

2H11 endothelial cells growing exponentially were plated in 6 –well culture plates at 3 x 10^5^ cells/well. Three washes with PBS were performed to remove serum prior to adding serum-free media. One row of wells (3 wells) was incubated with 75 μg/mL pimonidazole (equivalent to the *in vivo* dose regimen) in serum free media while the other row received serum free media and no pimonidazole. Hypoxic conditions were generated 24 hours after sub-culturing in 6-wells plates. Hypoxia was induced by incubating cells for 2 hours at 37°C in an anaerobic chamber (Forma Scientific Inc.) were a gas mix (5% CO_2_, 10% H_2_, 85% N_2_) produced oxygen concentrations within the tissue culture dishes at or below 10 mmHg. This was confirmed with flow cytometry by pimonidazole saturation of cells incubated under these conditions [23, 24].

Following hypoxia incubation with and without pimonidazole, 6-well plates were removed from the anaerobic chamber and the following procedure followed. All wells were washed with PBS (3X) followed by 1 mL of serum free media added with 10 μL of MBα-pimo. MBs were allowed to incubate with cells for 5 minutes, after which a gentle wash with serum free media and immediate phase-contrast imaging was performed on an Olympus IX71.

### Murine mammary gland carcinoma models

Two murine mammary gland carcinomas models (4T1, SCK) were generated in 5–12 week old female Balb/c and AJ mice (Charles River Laboratories) as described previously [20, 25]. MMTV-Wnt-1-Tg mice develop spontaneous mammary gland tumors and were kindly provided by Dr. Rosalia Simmen (UAMS). Subcutaneously implanted tumors in the right rear limb reached an approximate size of 200 mm^3^ in size by day 7, after which tumors underwent contrast-enhanced ultrasound imaging (as described below) and were excised for further histological studies. All animal experiments were performed with the approval of the University of Arkansas for Medical Sciences Animal Use and Care Committee (IACUC).

### Immunofluorescence

Mice received intraperitoneal (i.p.) injections with 75 mg/kg pimonidazole 2 hours prior to euthanasia and tissue collection, as described previously [20, 26]. Pimonidazole, a substituted 2-nitroimidazole (290.7 Da), is preferentially reduced in hypoxic viable cells and forms irreversible protein adducts, and has been optimized for detection with immunohistochemical and fluorescence methods [20, 26]. Normal (kidney, liver, and spleen) and tumor tissues were harvested and snap frozen in OCT for histological sectioning (5 μm) and subsequent staining. Hypoxia was detected by anti-pimonidazole antibody (mouse FITC-MAb 1:50, Hypoxyprobe, Inc.) and tumor blood vessels were detected by primary antibody CD31 (rat anti-CD31 1:100, BD Pharmingen) and secondary antibody anti-rat Alexa 647 (1:100, Invitrogen Molecular Probes) mounted in Vectashield mounting media with DAPI (Vector Labs, Inc.). Imaging was performed using an Olympus IX71 fluorescent microscope workstation or Aperio Scanscope FL.

### 2D High-frequency ultrasound imaging

Non-linear imaging of targeted, contrast-enhanced microbubbles within tumor tissue was performed according to the manufacturer’s protocol (FUJIFILM Visualsonics, Inc.). Two hours after intraperitoneal (i.p.) administration of 75 mg/kg pimonidazole (Hypoxyprobe, Inc), mice were anesthetized with 1% isoflurane and placed on a heated platform for the duration of the ultrasound imaging session. A 27G catheter was placed in the lateral tail vein and a 50 μL bolus of microbubbles injected intravenously (i.v.) using a syringe pump. After 5 minutes, allowing the microbubbles to circulate and bind, a programed data collection sequence was initiated and captured using a MS250 transducer, 18 MHz (FUJIFILM Visualsonics, Inc.). The program includes an initial data collection sequence during 25 seconds (designated ‘pre’), followed by a destruction phase where 100% of the microbubbles within the transducer field are obliterated by a high-mechanical index ultrasound pulse that eliminates both free and bound microbubbles, to complete with a 25 second data collection sequence of imaging after the burst (designated ‘post’). Comparisons were made between the steady state prior to and following the microbubble burst sequence. The difference in signal between pre- and post-burst (differential targeted expression, d.T.E.), represents the relative amount and location of microbubbles bound to pimonidazole. Four experimental conditions were investigated in the 4T1 tumor model: nontargeted-microbubbles which lack streptavidin moieties (MB) and pimonidazole-targeting MB (MBα-pimo) within the tumor and with circulating pimonidazole present, tumoral MBα-pimo without pimonidazole present (Fig 1A), and MBα-pimo measured within the muscle while circulating pimonidazole present (n = 3–11).

### 3D High-frequency ultrasound imaging, 3D modeling and animation

A modified manufacturer’s protocol was used to obtain 3D distribution of tumor vessel hypoxia. Five minutes after i.v. injection of targeted-microbubbles with a 27G 0.5cc insulin syringe (Terumo), 10 repetitive burst sequences were performed over the heart to destroy microbubbles in a highly concentrated region, until baseline contrast signal was observed. Following reduction of circulating unbound targeted-microbubbles, 3D imaging was acquired according to the manufacturer’s protocol. At 0.152mm/slice, 110 slices were collected from the tumor pre- and post-burst. Images were extracted and used for subsequent modeling and animation.

The unprocessed microbubble images collected with Visualsonics software were post-processed using a MatLab subtraction algorithm to illustrate the absolute signal difference between pre and post-burst. Deconvolved images were stacked with ImageJ software and used to create a 3D model and video. Additionally, Huygens Essential v2.10 software (Scientific Volume Imaging, B.V.) was used to further process deconvolved stacked images using maximum-intensity projections of the image stacks for 3D image modeling.

### Statistical Analysis

A one-way ANOVA with post-hoc Holm-Sidak’s multiple comparisons test was used for statistical analysis of microbubble data.

## Results and Discussion

### Pimonidazole targeting microbubbles

When pimonidazole is reduced within or on the surface of hypoxic cells, it forms an artificial tumor target [27] impervious to the tumor’s genetic origin or acquired mutations [19, 22], allowing broad-spectrum application. Here we designed a novel strategy using vascular restricted microbubbles to exploit this potential diagnostic and therapeutic target of vascular hypoxia (Fig 1). Targeting an artificially created target, the pimonidazole-targeting MB (MBα-pimo) can inherently act as its own control, i.e. in the absence of the target (Fig 1A). MBα-pimo can bind hypoxic endothelial cells *in vitro* incubated with pimonidazole, whereas the absence of pimonidazole prevents MBα-pimo binding (Fig 1B and S1 Fig).

### Vessel hypoxia detection

**Fig 2:**
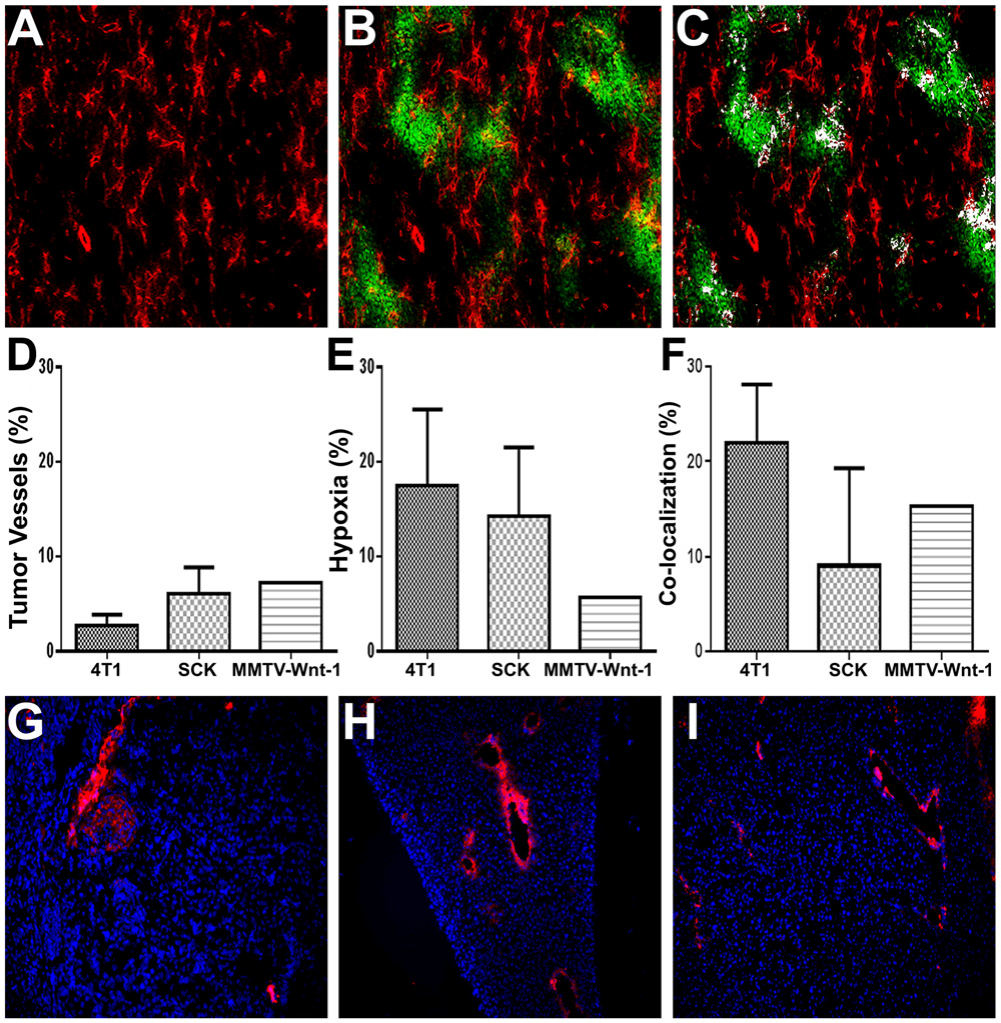
Vascular hypoxia in murine breast carcinomas and normal tissue. Immunofluorescence analysis of hypoxemia in 4T1 mammary gland carcinoma. **(A)**, 4T1 tumor tissue is stained for tumor vessels (CD31; red). (**B)** Tumor hypoxia (pimonidazole; green) is co-localized (white) in relation to microvasculature in 4T1 tumor tissue **(C)**. Quantification of overall tumor vessels **(D)**, hypoxia **(E)**, and hypoxic tumor vessels **(F)** in 4T1, SCK and MMTV-Wnt-1 carcinomas. Immunofluorescence analysis of vasculature (CD31; red) and hypoxia (pimonidazole; green) in non-diseased kidney **(G)**, spleen **(H)** and liver **(I)** indicates a lack of global and vessel hypoxia in normal tissue.

After demonstrating *in vitro* that MBα-pimo was specific to hypoxic endothelial cells incubated with pimonidazole, quantification of the amount of vascular hypoxia in three different murine mammary gland carcinoma models using immunohistochemical staining was performed, i.e. 4T1, SCK and MMTV-Wnt-1 (2). Whereas overall tumor hypoxia, as indicated by pimonidazole positive staining ranged from 5%– 18% in these models, vascular hypoxia, as indicated by co-localization of tumor vessels (CD31^+^) and hypoxia (pimonidazole^+^) amounted up to 20% of the total stained vasculature in the 4T1 model (Fig 2F). These findings are similar to earlier reports studying various malignant tissues and hypoxia subtypes [28]. In contrast, no vascular hypoxia was detected in normal healthy tissues i.e. kidney, spleen and liver (Fig 2G, 2H and 2I); suggesting cell surface pimonidazole targets are a promising hypoxia target in malignant tissues (S2 Fig).

### High-frequency ultrasound imaging with pimonidazole-targeted microbubbles

**Fig 3:**
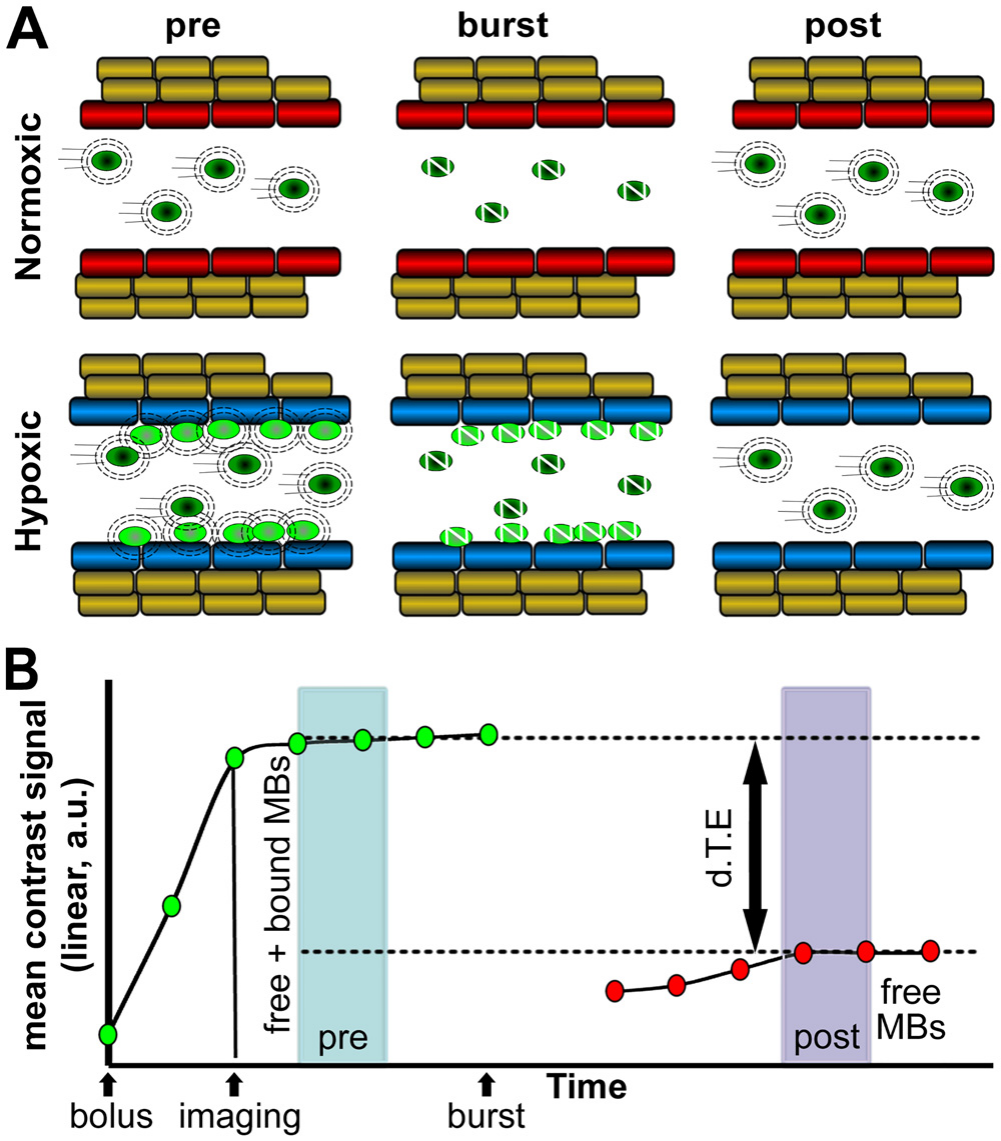
Schematic of anti-pimonidazole targeted-microbubbles (MBα-pimo) with ultrasound imaging for detection of vascular hypoxia. **(A)** Illustration showing the differential distribution of MBα-pimo in well-oxygenated tumor endothelium (red) compared to hypoxic tumor endothelium (blue) during imaging and intervention by ultrasound. **(B)** Representative quantification graphic of MBα-pimo where the binding occurs over a 5 minute window after IV injection followed by a data collection period of contrast signal, a single ultrasound pulse to burst bound and free MBα-pimo, and a final data collection during the immediate reperfusion window. Subsequently, the difference in signal from the steady state prior to microbubble burst (‘pre’) and following burst (‘post’) can be calculated. This differential targeted expression (d.T.E.; linear, a.u.) represents the relative amount of bound MBα-pimo and indirectly indicates the location and amount of vascular hypoxia within the tumor (x-axis scale not linear).

High-frequency ultrasound was used to image and quantify bound pimonidazole-targeted microbubbles (MBα-pimo) in tumor and normal tissue (Fig 3).

**Fig 4:**
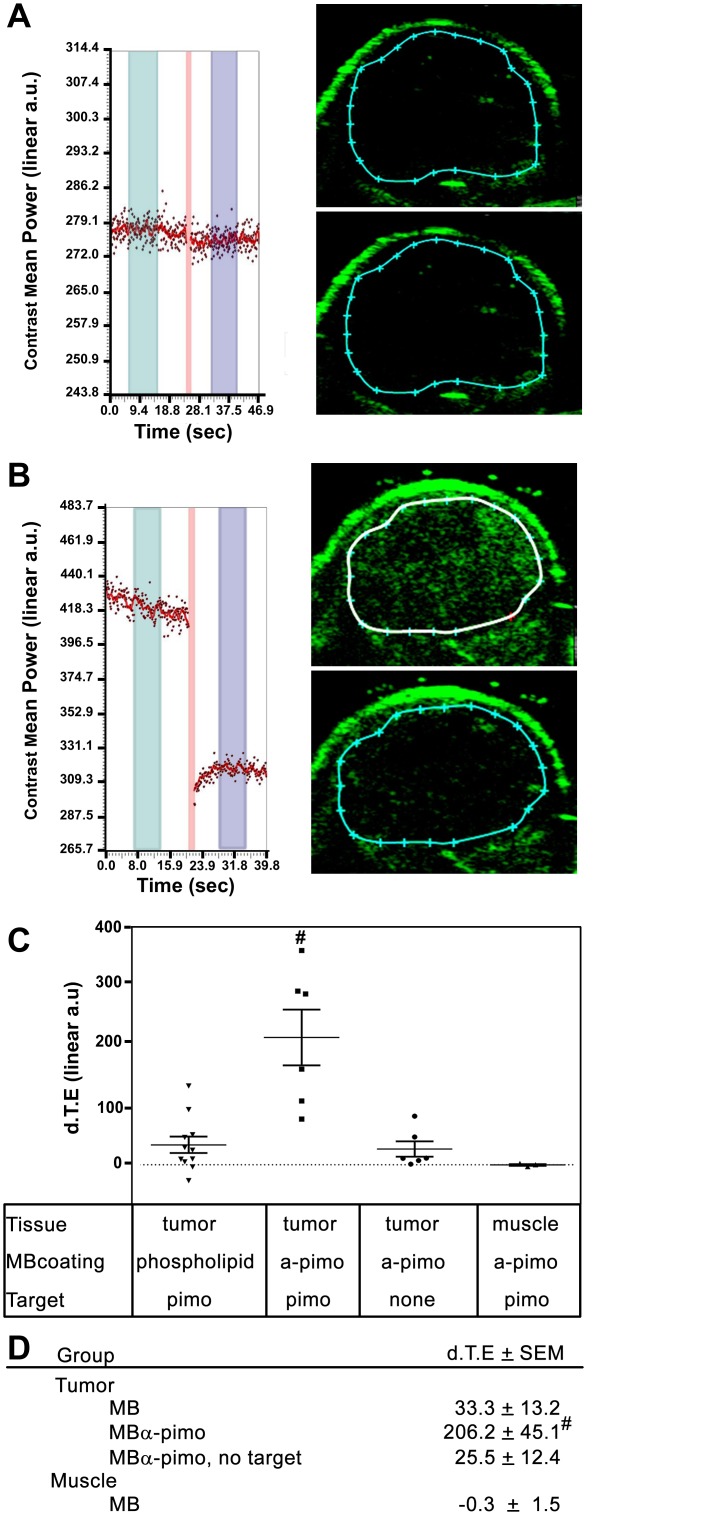
High-frequency ultrasound imaging of targeted-microbubbles detects tumor vessel hypoxia. Representative image and quantified data of anti-pimonidazole labeled microbubbles (MBα-pimo) bound in perfused hypoxic tumor vasculature without pimonidazole injection **(A)**, and with pimonidazole injection **(B)** in 4T1 tumor bearing mice. Top image shows the signal before the burst sequence and the bottom image shows after the burst sequence **(A, B)**. **(C)** Quantified data of different experimental conditions using targeting and non-targeting microbubbles (as indicated). **D)** Summary of quantitated data statistically analyzed represented as mean ± SEM, ^#^p < 0.05, versus non-targeting MB, MBα-pimo without pimonidazole injection, and MBα-pimo in muscle tissue (ANOVA post-hoc Holm-Sidak).

By creating the artificial targets with injection of pimonidazole, the accumulation of MBα-pimo contrast agent in tumor vessels was significantly enhanced with a nearly 8-fold higher differential targeted expression (d.T.E.) (206.2 ± 45.1 vs. 25.5 ± 12.4 linear a.u., p < 0.05) than in tumors not receiving pimonidazole. In normal healthy muscle tissue, the pre- and post- signal was not significantly different resulting in a d.T.E. of essentially zero (-0.33 ± 1.4 linear a.u.) despite the presence of circulating pimonidazole. Non-targeted-microbubbles (MB) accumulated within the tumor similar to controls (MBα-pimo in mice lacking pimonidazole injection), however with greater variance (33.3 ± 13.2 linear a.u., Fig 4C and 4D). Passive accumulation and extravasation into the tumor might contribute to this, although this effect should be inherent in the other conditions and would require inter-endothelial cell junctions much larger than typically reported [29]. Relatedly, high interstitial fluid pressure, which is a well-documented physiological feature of solid tumors [30–33], might affect contrast-enhanced ultrasound imaging with microbubbles. The high intra-tumoral pressure can lead to reduced tumor blood flow and thus, may influence microbubble imaging through dynamic changes in blood flow kinetics [21]. Reduced or sluggish blood flow may also result in trapping or non-specific binding/electrostatic interactions within tumor blood vessels. We were cognizant that other external factors may also exacerbate the influence on physiology (i.e. anesthesia, body temperature) and contribute to the amount of non-specific signal. Nonetheless, the signal generated in the tumor from targeted microbubbles suggests a detectable level of hypoxic vasculature is present in these models and may be translated into human cancer.

**Fig 5:**
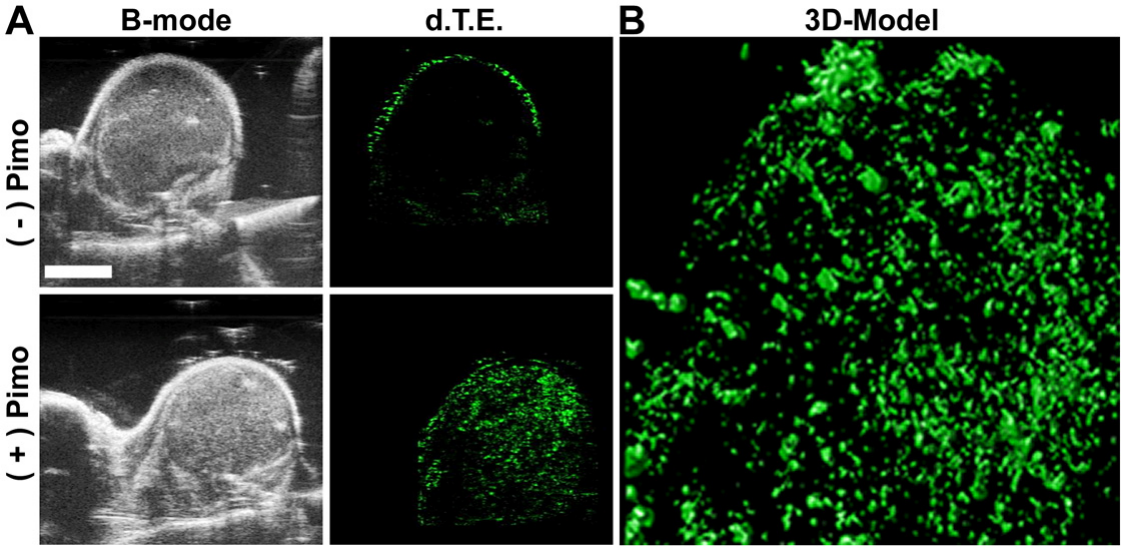
3D modeling of MBα-pimo distribution in mammary gland carcinoma. **A)** Single slice images taken from a 3D imaging sequence in B-mode *(left)*. Single slice images taken from a 3D imaging sequence depicting the differential targeted expression (d.T.E) *(right)*. **B)** Three-dimensional contrast projection of 3D stack image data from hypoxia targeted, MBα-pimo, contrast signal collected in a rear-limb 4T1 tumor. Images (0.152mm/slice) generated using Visualsonics imaging system and post-processed using the Huygens essential software.

While this molecular imaging methodology allows for imaging of tumor vessel hypoxia in a specific 2D plane, it also permits imaging of the whole tumor. By using specialized software (Huygens essential software), a 3D representation of overall vessel hypoxia was generated (Fig 5B). This permits not only a read-out on the general hypoxic state of the tumor, but also whether there is micro-regional hypoxia within the tumor, i.e. improved resolution over standard hypoxia imaging techniques. Prior to non-linear contrast-enhanced imaging, anatomical visualization was obtained with the B-mode ultrasound (Fig 5A) followed by imaging of MBα-pimo in 3D. Following image collection, post-processed images reflect the d.T.E (Fig 5A). Subsequently, the deconvolved images were stacked to build a maximum-intensity 3D model and video (Fig 5B and S3 Fig). The ability to image whole tumor hypoxemia presents an attractive method for longitudinal, clinically meaningful studies on its role in tumor progression and therapeutic response. For example, hypoxia has attenuating anti-tumor effects on radiotherapy and drug efficacy [12–14], and while these tempering effects have been reported for various tumor and normal cell types [16], there is a paucity of investigations delineating the direct consequences of hypoxia on radiation or drug efficacy in tumor endothelial cells, *in vitro* or *in vivo*. Conversely, there is a substantial amount of literature identifying the vascular condition in response to various treatment therapies. The detection of vascular hypoxia using contrast-enhanced ultrasound as described within would permit a directed and non-invasively applied strategy for pre-clinical and ultimately clinical settings focused on the status of the vasculature before, during or after therapy.

## Conclusions

Studies have shown that hypoxic vessels are associated with tumor angiogenesis, progression, stem cell induction/protection, metastasis and possibly revascularization of recurrent tumors [4, 6–10, 34]. The realization that hypoxic microvasculature exists as early as the developmental stage [4, 5] and plays a key-role in tumor progression and treatment response, brings about new possibilities for the role of hypoxia in cancer biology. Improved radiation delivery techniques allow the potential for dose painting tumor areas found to harbor hypoxic tumor vessels. In addition, creating a new target with an exogenously administered agent of high specificity to the tumor vasculature and tumor microenvironment (e.g. hypoxia) presents a unique and ideal platform for drug delivery to tumor and stromal cells via antibody directed drug conjugates, targeted liposomes and other targeted drug-encapsulated technologies [27]. While some “non-specific” binding with non-targeted-microbubbles were noted in this approach, further study and refinement of the microbubble imaging technique should provide insight into the underlying variables involved and could potentially reveal further applications of this imaging technique if correlated with tumor IFP or other diagnostic tumor physiology [32, 33]. The data presented herein suggests tumor vessel hypoxia is a detectable physiological phenomenon utilizing a new method of pimonidazole-targeted contrast-enhanced microbubbles confined to the microcirculation of a tissue. Segmenting subtypes of hypoxia with an imaging technique that possess capillary level resolution, provides exciting new opportunities for longitudinal studies investigating vascular hypoxia in tumor progression and therapeutic response. The method described is based on generating artificial targets able to be ‘created’ on-demand by injection or ingesting pimonidazole, an already clinical approved agent. Thus, this method does not rely on heterogeneously or transiently expressed biomarkers of any kind, is not affected by genetic variability or constrained to the tumor microenvironment; instead, it is a snapshot of hypoxia occurring in and around vasculature in a variety of ischemic conditions.

## Supporting Information

S1 Fig Anti-pimonidazole antibody binds cell surface pimonidazole antigens in the presence of hypoxia. MBα-pimo does not bind endothelial cells without pimonidazole (*left* vs. *right panels*), and hypoxic conditions (*top vs*. *bottom panels*).
(TIF)

S2 Fig Evaluation of cell surface anti-pimonidazole antigens by flow cytometry. Pimonidazole adduct formation on the cell membrane is preferentially formed under hypoxic conditions. Cells negative for 7-AAD, a fluorescent compound with a strong affinity for DNA were deemed viable and non-porous, thus preventing any significant degree of intracellular binding of anti-pimonidazole antibody.
(TIF)

S3 Fig 3D video of vascular hypoxia after off-site bursting all free microbubbles in circulation: detection of anti-pimonidazole functionalized microbubbles. A 3D video was made using a customized Matlab algorithm to subtract the free flowing microbubble signal in order to display the relative amount and distribution of MBα-pimo, or tumor vessel hypoxia. Representative control (no pimonidazole) and pimonidazole-injected tumor bearing mice imaged with MBα-pimo are shown. A static B-mode image from the central region of the tumor is shown for orientation.
(PPTX)
