# Supplementary figures and images for: Targeting Artificial Tumor Stromal Targets for Molecular Imaging of Tumor Vascular Hypoxia
Source: PLoS One. 2015 Aug 26;10(8):e0135607. doi: 10.1371/journal.pone.0135607 (PMC4550408; doi:10.1371/journal.pone.0135607)

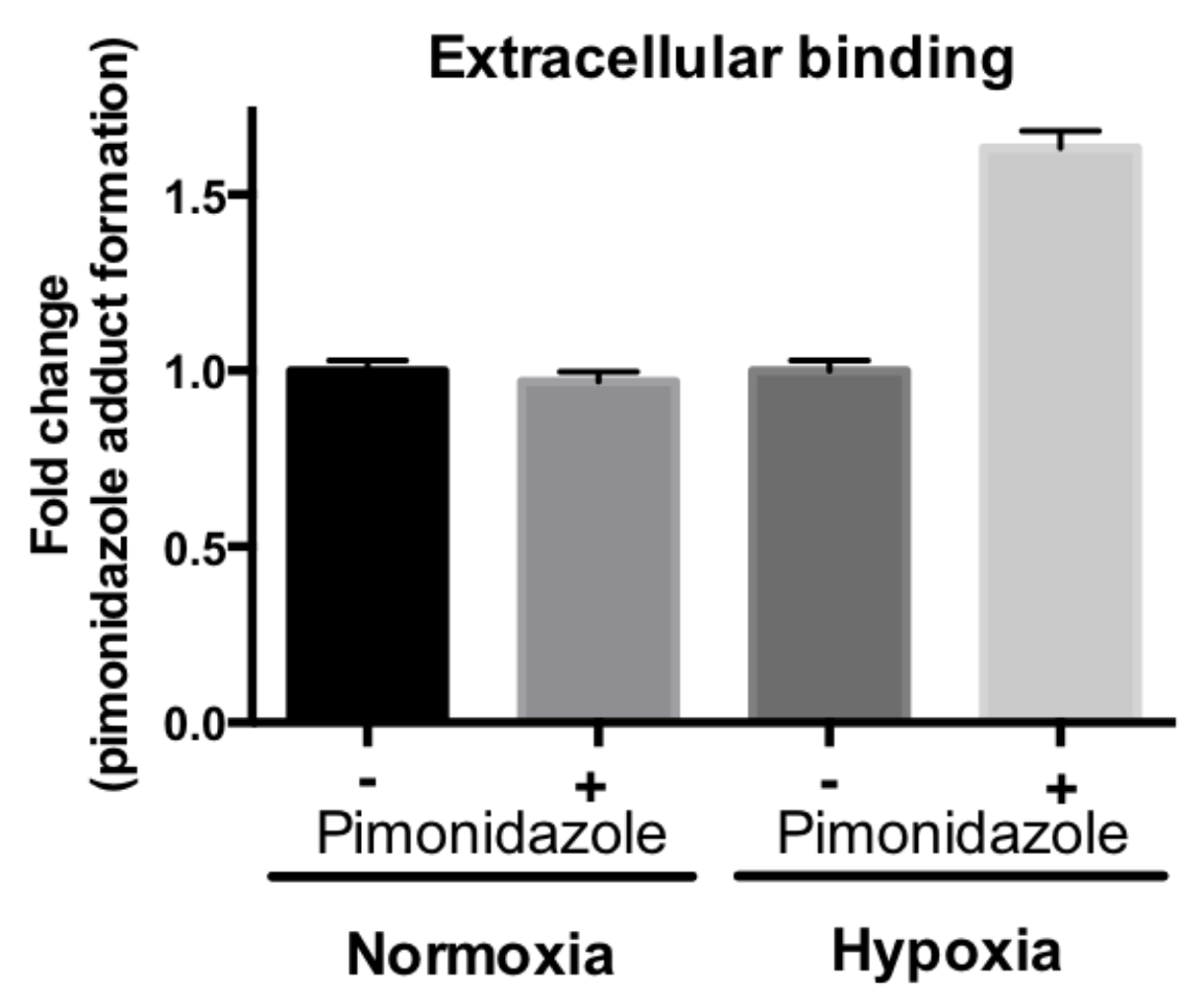

Supplement: S2 Fig — Pimonidazole adduct formation on the cell membrane is preferentially formed under hypoxic conditions. Cells negative for 7-AAD, a fluorescent compound with a strong affinity for DNA were deemed viable and non-porous, thus preventing any significant degree of intracellular binding of anti-pimonidazole antibody. (TIF) [file pone.0135607.s002.tif]

## Slide 1
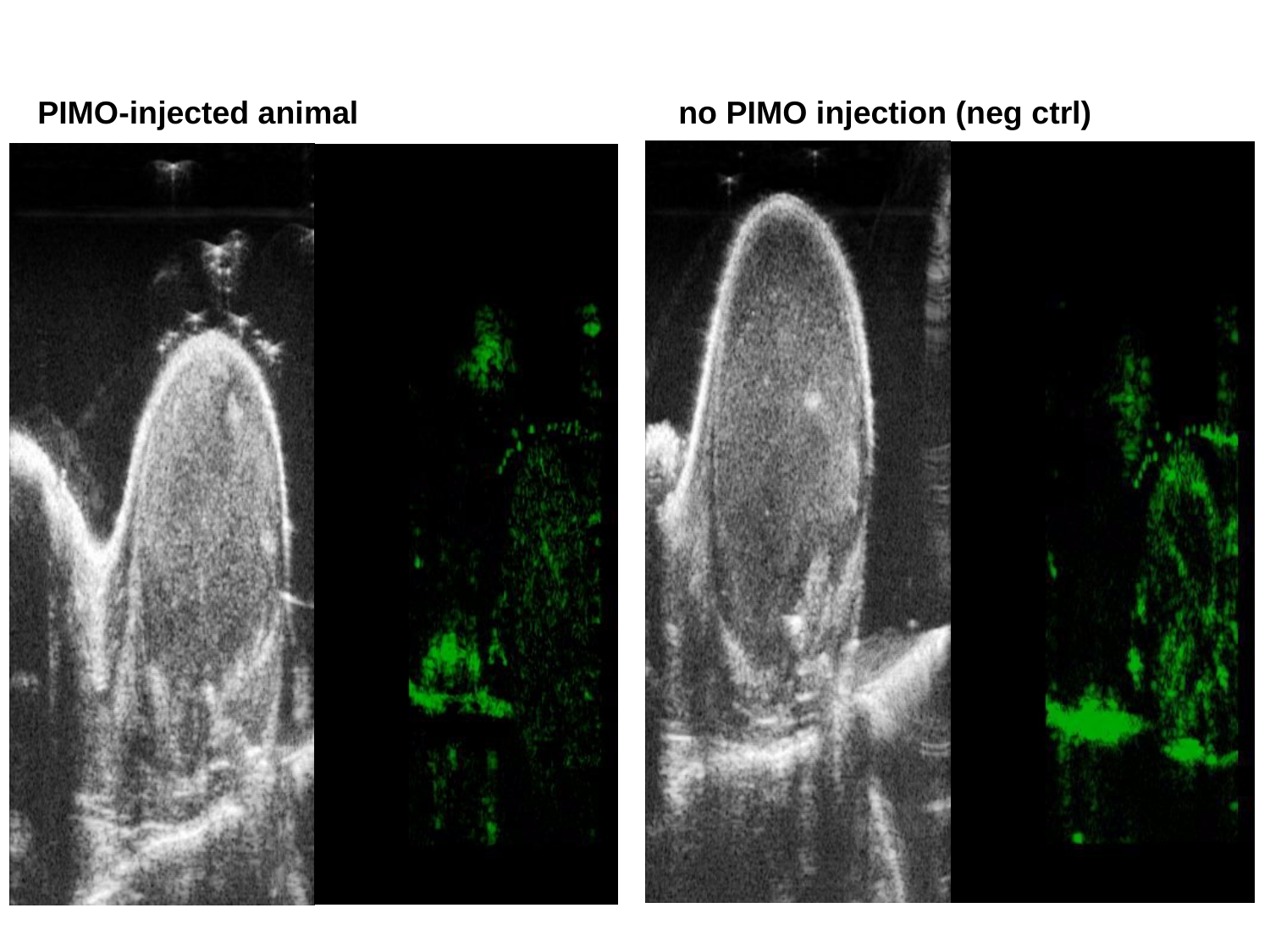

PIMO-injected animal		 no PIMO injection (neg ctrl)

Supplement: S3 Fig — A 3D video was made using a customized Matlab algorithm to subtract the free flowing microbubble signal in order to display the relative amount and distribution of MBα-pimo, or tumor vessel hypoxia. Representative control (no pimonidazole) and pimonidazole-injected tumor bearing mice imaged with MBα-pimo are shown. A static B-mode image from the central region of the tumor is shown for orientation. (PPTX) [file pone.0135607.s003.pptx]
